# Supplementary material for: Connecting genomic results for psychiatric disorders to human brain cell types and regions reveals convergence with functional connectivity
Source: Nat Commun. 2025 Jan 4;16:395. doi: 10.1038/s41467-024-55611-1 (PMC11700164; doi:10.1038/s41467-024-55611-1)
Supplement: Supplementary file 1 — Supplementary Information [file 41467_2024_55611_MOESM1_ESM.pdf]

Supplementary Information

Connecting genomic results for psychiatric disorders to human brain cell types and regions reveals convergence with functional connectivity

Shuyang Yao[1,2], Arvid Harder*[1,2], Fahimeh Darki*[3], Yu-Wei Chang[4], Ang Li[5], Kasra Nikouei[1], Giovanni Volpe[4], Johan N Lundström[3,6], Jian Zeng[5], Naomi R. Wray[5,7], Yi Lu[2], Patrick F Sullivan[†2,8], Jens Hjerling-Leffler[†1]

Author affiliations:
1. Department of Medical Biochemistry and Biophysics, Karolinska Institutet, Stockholm, Sweden
2. Department of Medical Epidemiology and Biostatistics, Karolinska Institutet, Stockholm, Sweden
3. Department of Clinical Neuroscience, Karolinska Institutet, Stockholm, Sweden
4. Department of Physics, University of Gothenburg, Gothenburg, Sweden
5. Institute for Molecular Bioscience, University of Queensland, Brisbane, Australia
6. Monell Chemical Senses Center, Philadelphia, PA, USA
7. Department of Psychiatry, University of Oxford, Oxford, UK
8. Departments of Genetics and Psychiatry, University of North Carolina, Chapel Hill, NC, USA

* Equal contributions. † Co-corresponding authors (jens.hjerling-leffler@ki.se and pfsulliv@med.unc.edu)

This PDF file includes 9 Supplementary figures and the member lists of consortia collaborators.

Source data of the main and supplementary figures are provided in the Source Data file.

Supplementary Figures

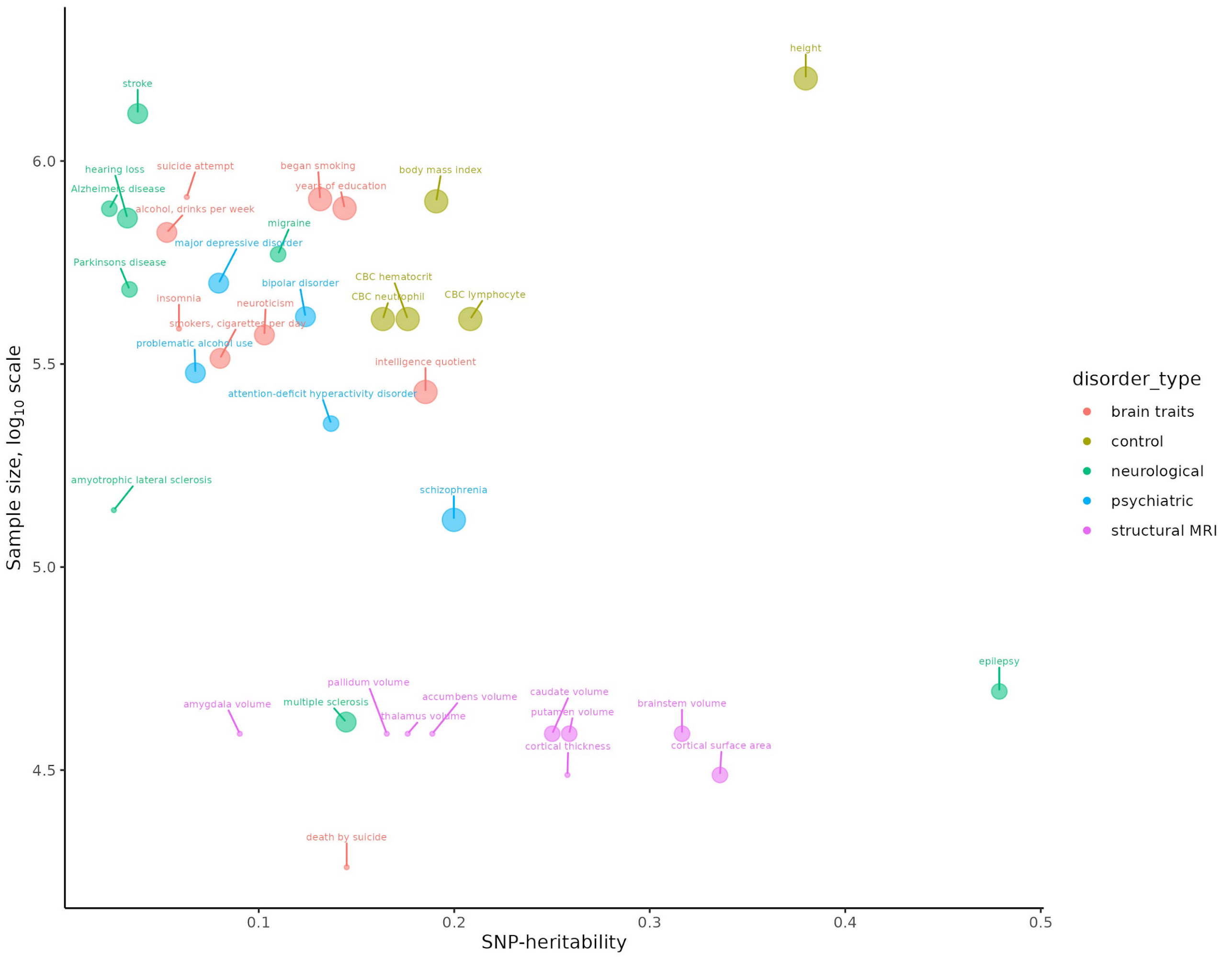

Figure S1. Depiction of primary GWAS trait features (data in Table S1). X-axis depicts liability-scale SNP-heritability estimated using LDSC. Y-axis is sample size on log_{10} scale. Point colors correspond to disorder type. Point size shows quartiles of the number of LD-clumped loci: quartile 1 had 3-13 loci (e.g., insomnia, suicide attempt); quartile 2 had 13-37 loci (e.g., Alzheimer's, epilepsy); quartile 3 had 40-103 loci (e.g., bipolar disorder, MDD); and quartile 4 had 180-2705 loci (BMI, educational attainment, height).

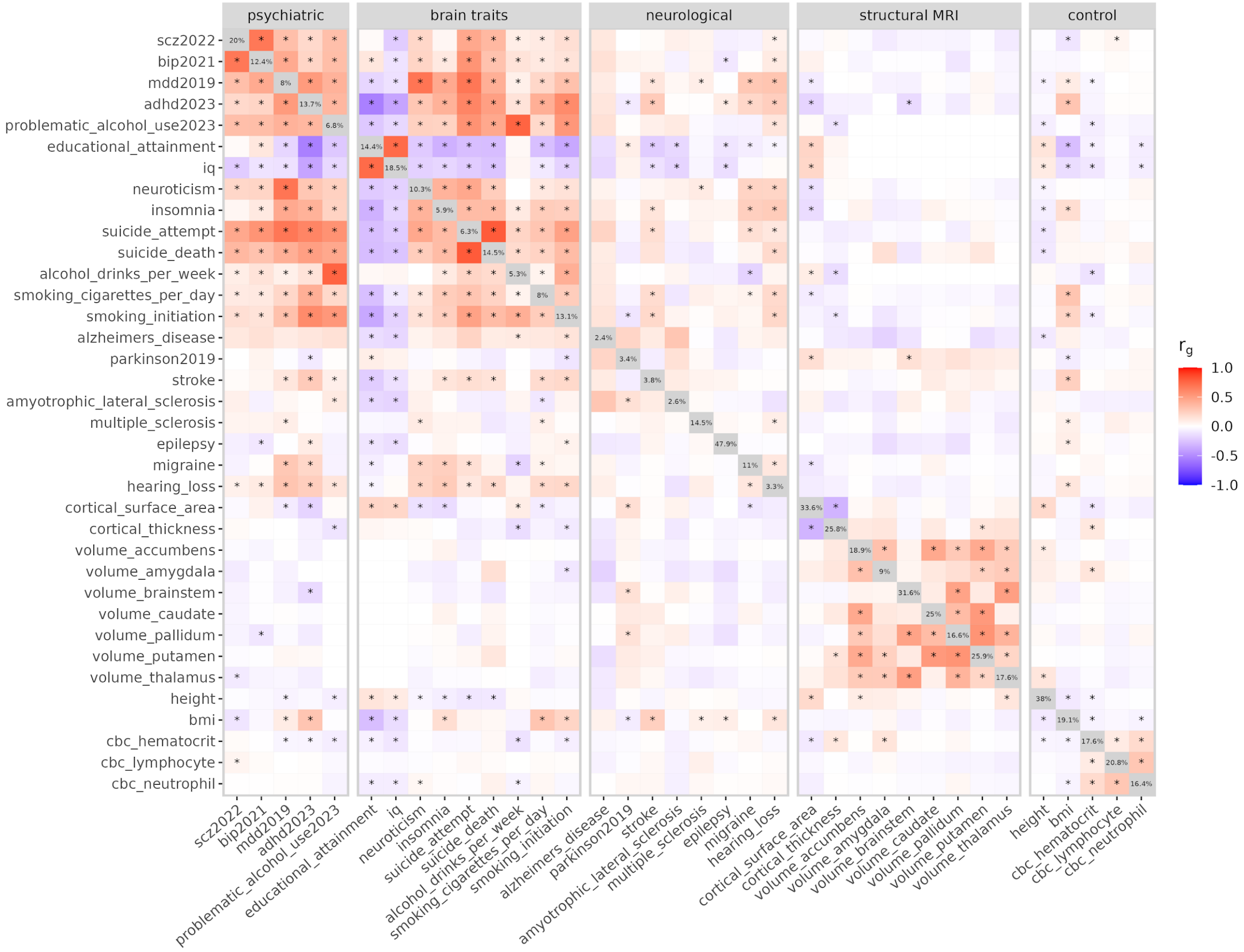

Figure S2. Heatmap of genetic correlations (r_g) for the primary GWAS traits. Trait labels are defined in Table S1. We used LDSC to estimate SNP-heritability of each trait (shown on the diagonal) and genetic correlations between traits (off-diagonal values). Traits are grouped by disorder type. Colors indicate the strength and direction of genetic correlations. Asterisks indicate significant genetic correlation at FDR < 0.05 per trait. We have blanked out the genetic correlations of subcortical volumes with IQ, educational attainment, and alcohol use (the data owners prohibit use for "research into the genetics of intelligence, education, … or addictions").

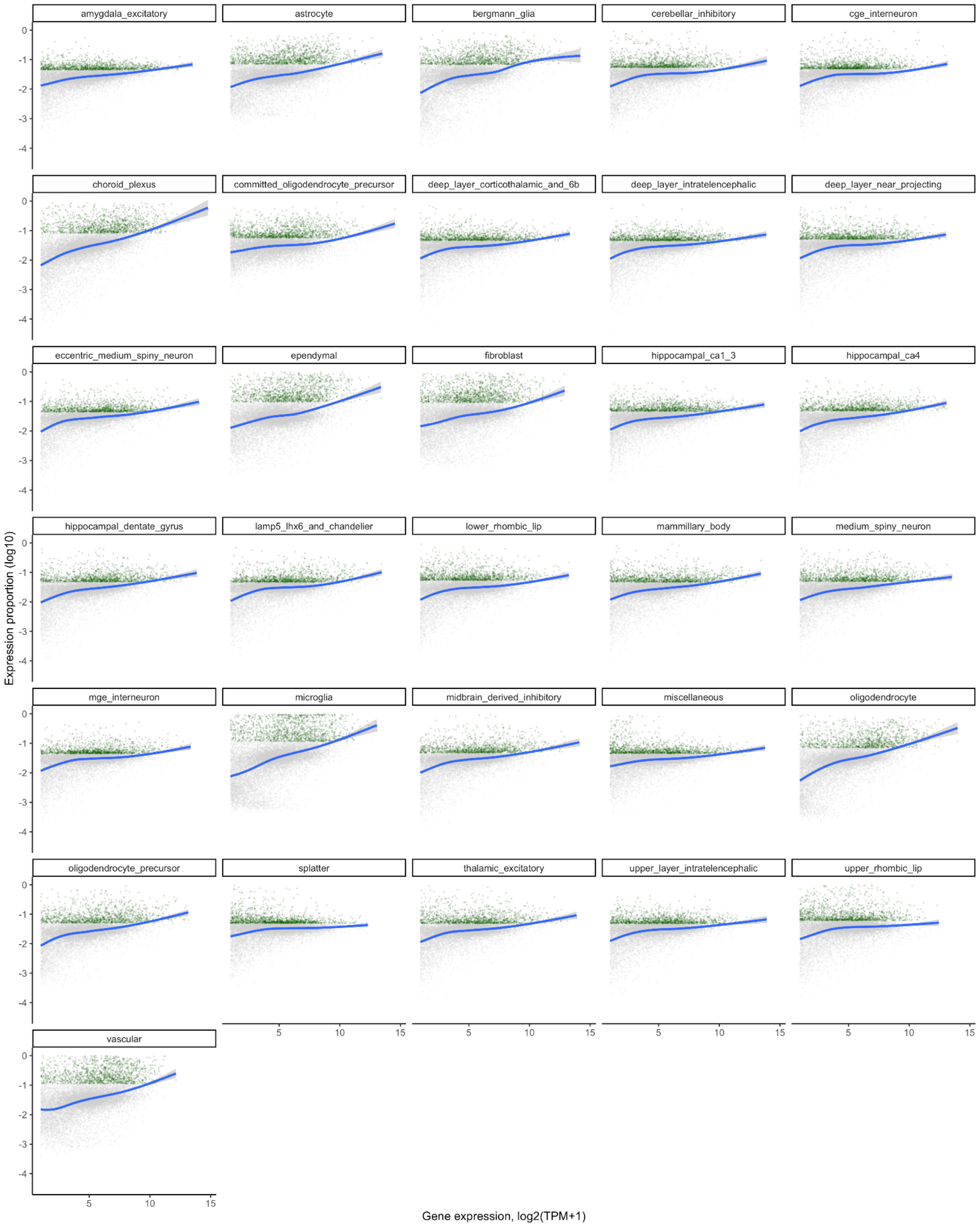

Figure S3. Relation of TPM (x-axis) and expression proportion (EP, y-axis) separately for each of the 31 superclusters. Both axes were log transformed to add separation. Each point is a brain-expressed protein-coding gene. Green points show the top-decile EP (TDEP) genes for a supercluster, and gray all other genes. The blue line is a lowess smoother.

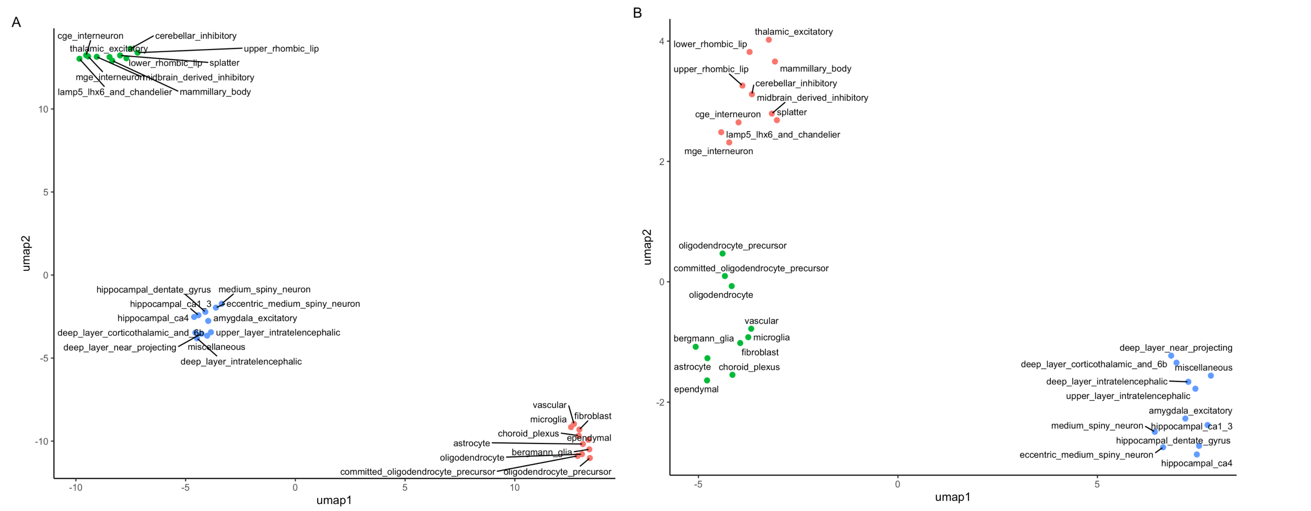

Figure S4. UMAP (n_neighbors=5) / HDBSCAN (minPts = 5) analysis. Panel A is for TDEP and panel B for $\log_2$(TMP+1).

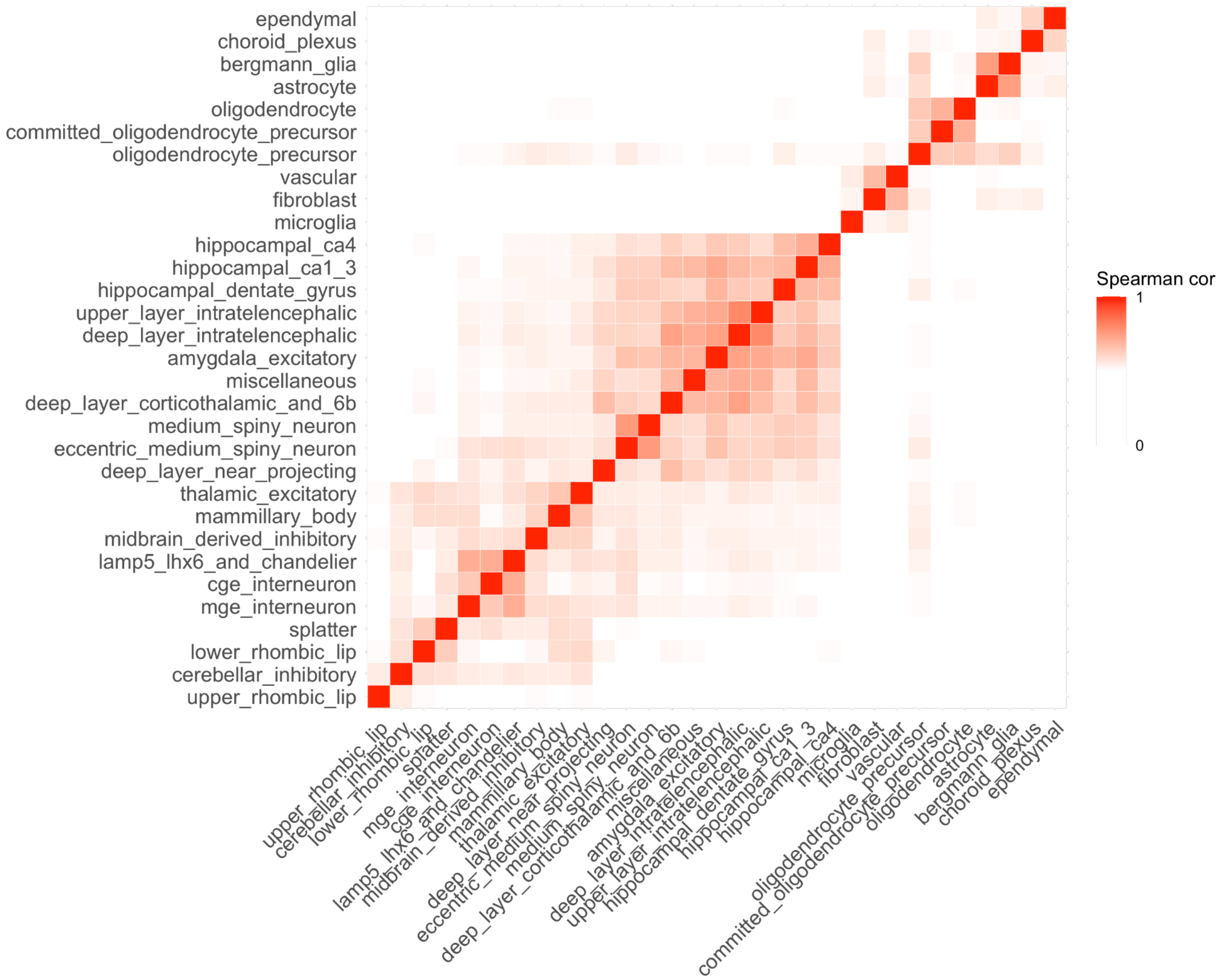

Figure S5. Genomic bin analysis. For each supercluster and 100 kg genomic bins, we numerically assessed the degree to which the observed number of TDEP gene TSS per bin deviated from the total number of protein-coding TSS per bin (studentized residuals). The above is a heatmap of the Spearman correlation matrix (following hierarchical clustering).

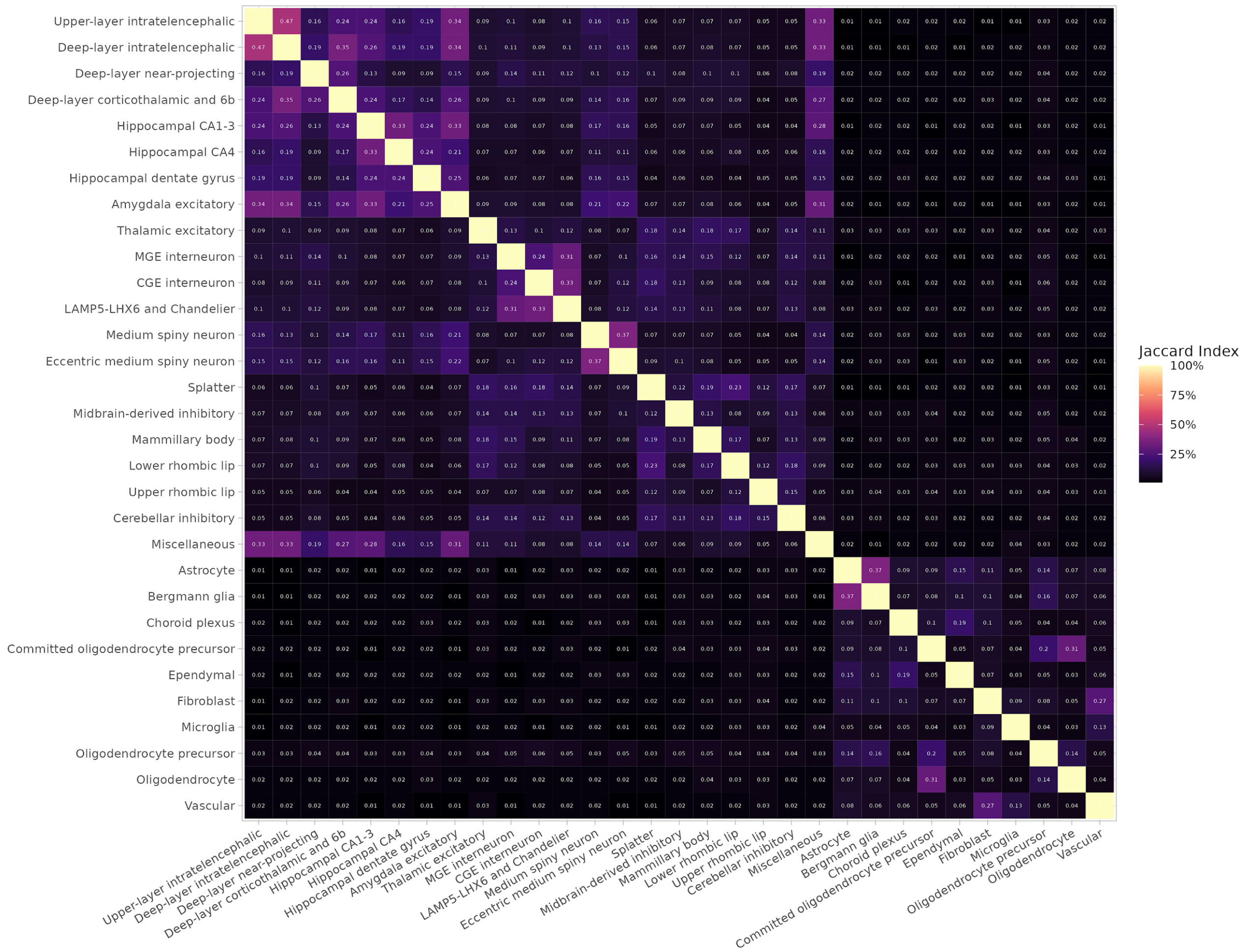

Figure S6. Jaccard index heatmap. For all pairs of superclusters, we computed the Jaccard index for overlap of TDEP genes (these gene sets are the input annotation for S-LDSC). For two sets of genes, Jaccard index of 1 means complete overlap and 0 means no overlap.

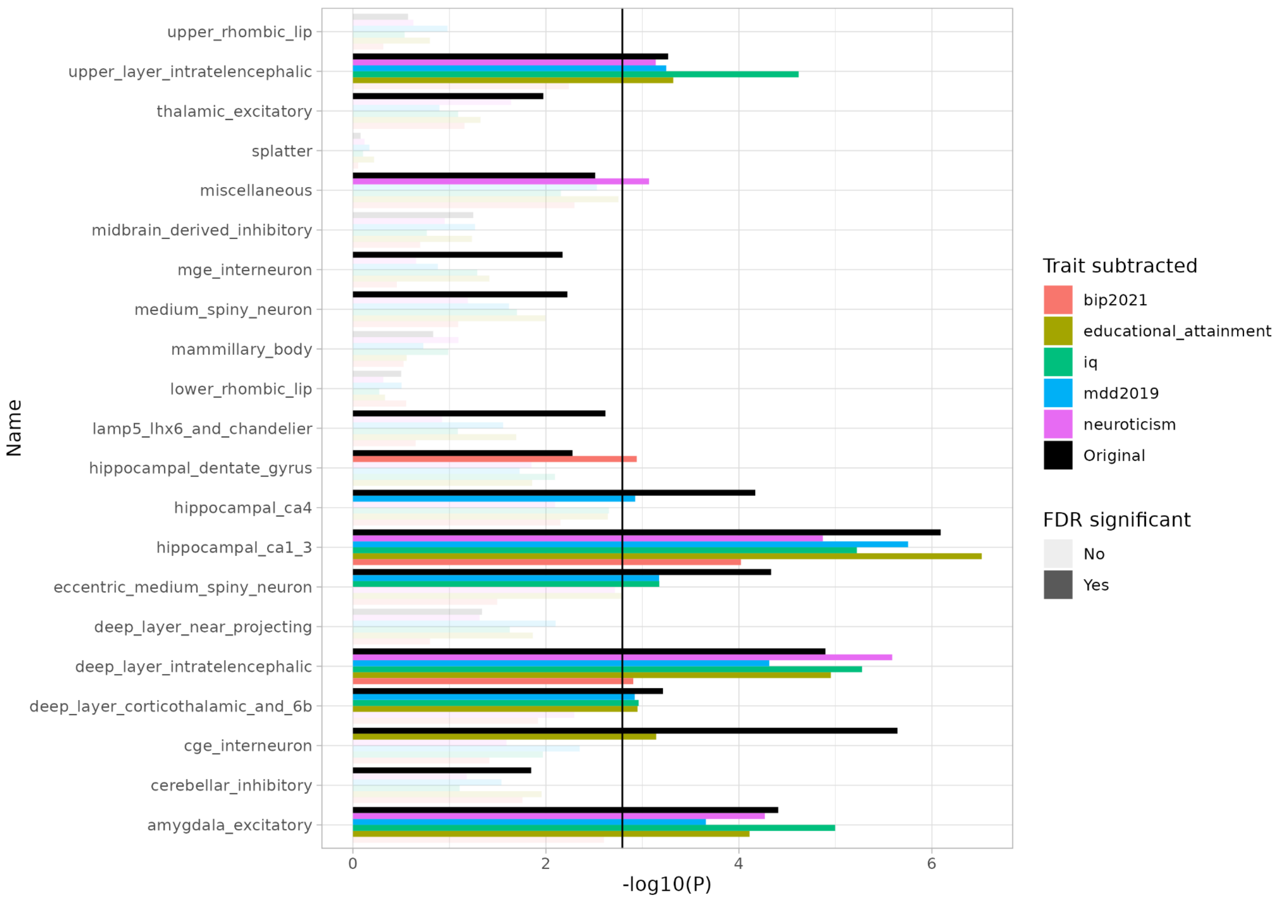

Figure S7. Superclusters enriched with schizophrenia-specific genetic risk, conditioned on bipolar disorder, MDD, IQ, neuroticism, and educational attainment; the opacity indicates significance according to FDR ≤0.05. The black bar indicates the original results (same as that for scz2022 in Figure 2A), and the other colors indicate the results conditioned on the corresponding trait. The line indicates Bonferroni threshold for P=0.05/31 tests

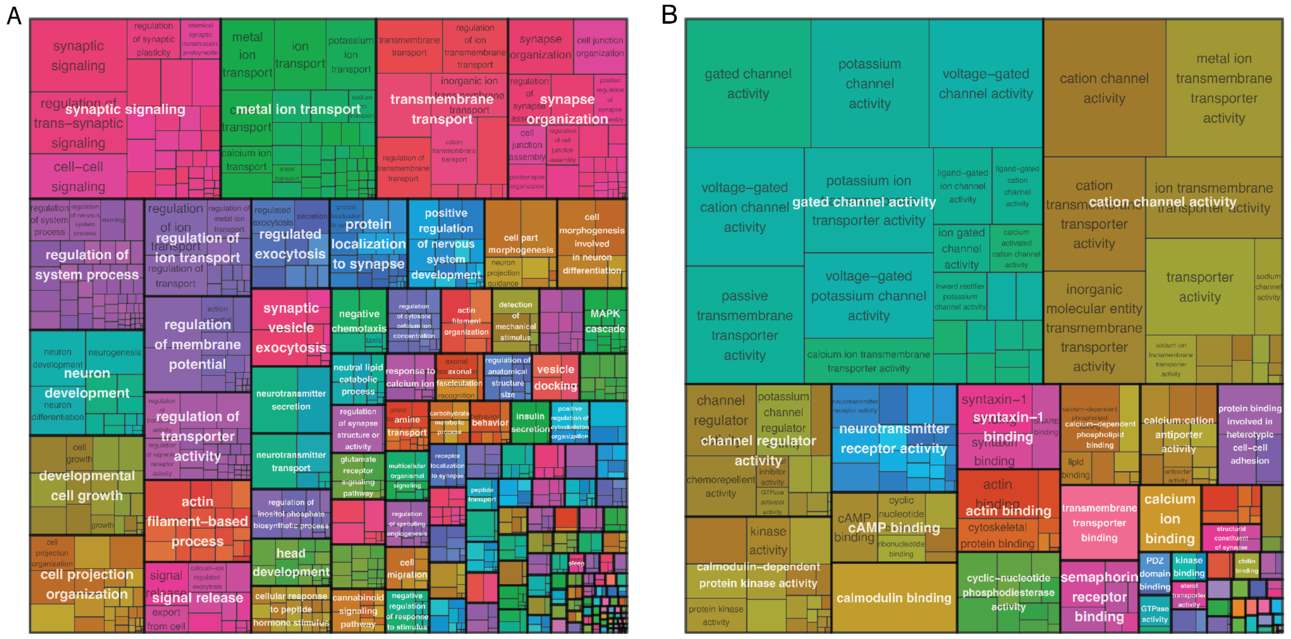

Figure S8. Treemap for gene ontology (GO) gene set enrichment for the TDEP genes of the top 25 clusters enriched of schizophrenia SNP-heritability. (A) GO-BP gene sets enriched in the clusters. (B) GO-MF gene sets enriched in the clusters.

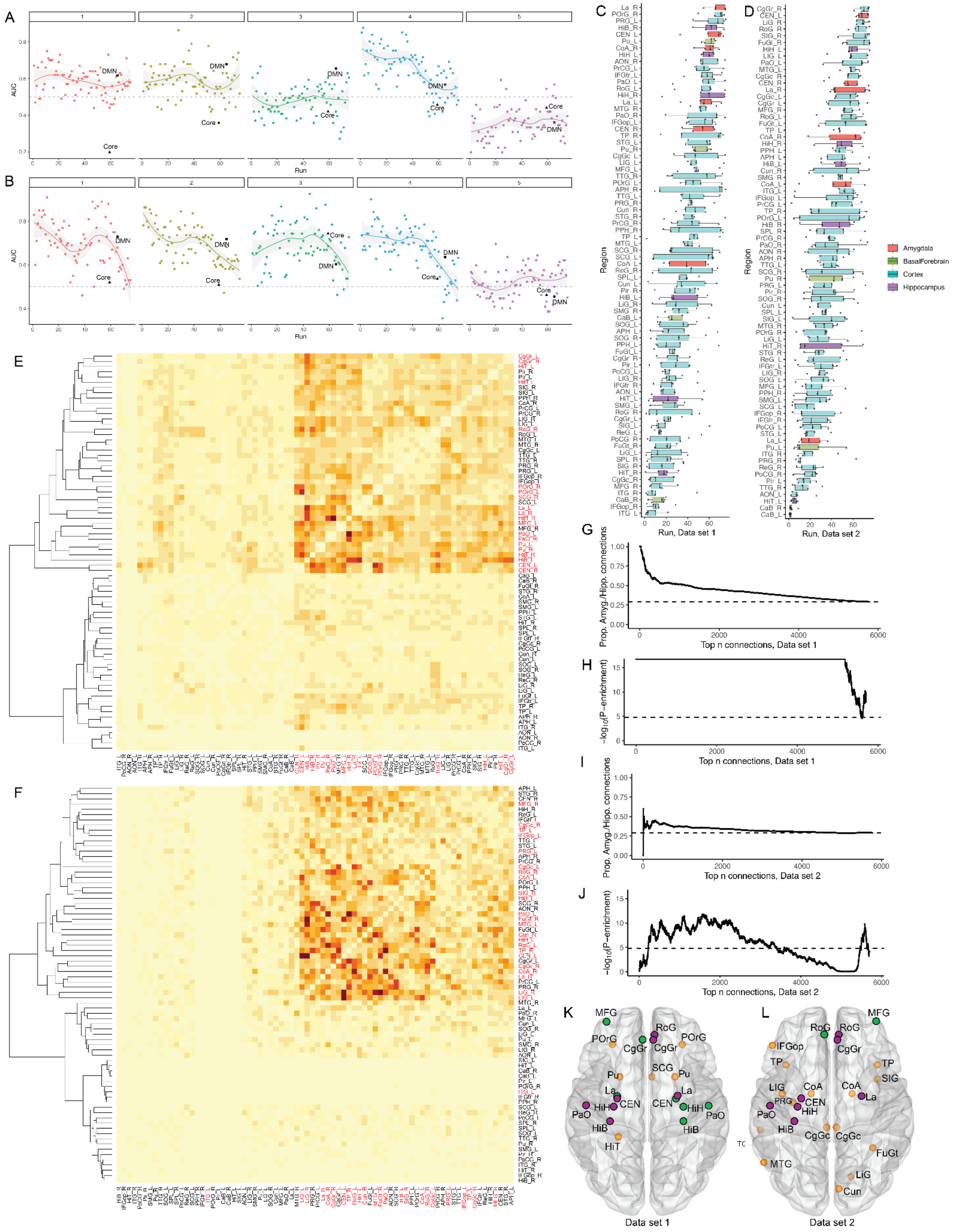

Figure S9. fMRI analysis supplementary figures. (A-B) The AUC of each model across the five parallel folds of recursive feature elimination in data sets 1 (A) and 2 (B). The AUCs of the default mode network (DMN, black squares) and core network (black triangles) were presented as a reference. (C-D) Regions of interest (ROIs) ranked by the preserved runs per fold in data sets 1

(C) and 2 (D). Each region had five values per data set as there were five parallel folds. Colors indicate the broader region the ROI came from (same as in Figure 5B). (E-F) The aggregated FI matrix in data sets 1 (E) and 2 (F). Darker cells indicate higher aggregated FI value of the corresponding connections. The ROIs were organized by hierarchical clustering of their connections (FI values), and the name was colored red if the ROI was involved in the top 1% connections; those ROIs were also plotted in panels K-L. (G, I) The proportion of amygdalar and/or hippocampal connections (y-axis) in the top n connections (x-axis), in data sets 1 (G) and 2 (I). (H, J) The significance, -log10(P), of enrichment of amygdalar and/or hippocampal connections (y-axis) in the top n connections (x-axis), in data sets 1 (H) and 2 (J); n was the even numbers from 2 to 5776, i.e. we performed 2888 hypergeometric tests per data set. The dashed line indicates the bonferroni corrected p-value, i.e., 0.05/2888 on -log10 scale. (K, L) Dorsal view of the ROIs of the top 1% connections in data sets 1 (K) and 2 (L). Purple dots suggest the same ROIs on the same hemisphere in the two data sets, green dots suggest the same ROIs on the opposite hemispheres in the two data sets, and yellow dots are ROIs different between the two data sets. Annotations of the ROIs are detailed in Table S11.

International Suicide Genetics Consortium

Anna R Docherty[1,2,3], Niamh Mullins[4,5], Allison E Ashley-Koch[6], Xuejun Qin[6], Jonathan R I Coleman[7,8], Andrey Shabalin[1,2], JooEun Kang[9], Balasz Murnyak[1,2], Frank Wendt[10], Mark Adams[11], Adrian I Campos[12,13], Emily DiBlasi[1,2], Janice M Fullerton[14,15], Henry R Kranzler[16,17], Amanda Bakian[2], Eric T Monson[2], Miguel E Rentería[12,18], Consuelo Walss-Bass[19], Ole A Andreassen[20,21], Cynthia M Bulik[22,23,24], Howard J Edenberg[25,26], Ronald C Kessler[27], J John Mann[28], John I Nurnberger Jr[29,29], Giorgio Pistis[30], Fabian Streit[31], Robert J Ursano[32], Renato Polimonti[10], Michelle Dennis[33], Melanie Garrett[34], Lauren Hair[35], Philip Harvey[36], Elizabeth R Hauser[6,37], Michael A Hauser[6], Jennifer Huffman[38], Daniel Jacobson[39], Jennifer H Lindquist[40], Ravi Madduri[41], Benjamin McMahon[42], David W Oslin[43,44], Jodie Trafton[45], Swapnil Awasthi[46], Andrew W Bergen[47,48], Wade H Berrettini[49], Martin Bohus[50], Harry Brandt[51,52], Xiao Chang[53], Hsi-Chung Chen[54], Wei J Chen[54,55,56], Erik D Christensen[57,58], Steven Crawford[51,52], Scott Crow[59], Philibert Duriez[60,61], Alexis C Edwards[3], Fernando Fernández-Aranda[62], Manfred M Fichter[63,64], Hanga Galfalvy[65,66], Steven Gallinger[67], Michael Gandal[68], Philip Gorwood[60,61], Yiran Guo[53], Jonathan D Hafferty[11], Hakon Hakonarson[53,69], Katherine A Halmi[70], Akitoyo Hishimoto[71], Sonia Jain[72], Stéphane Jamain[73], Susana Jiménez-Murcia[62], Craig Johnson[74], Allan S Kaplan[75,76,77], Walter H Kaye[78], Pamela K Keel[79], James L Kennedy[75,76,77], Minsoo Kim[68], Kelly L Klump[80], Daniel F Levey[81,82], Dong Li[53], Shih-Cheng Liao[54], Klaus Lieb[83], Lisa Lilenfeld[84], Adriana Lori[85], Pierre J Magistretti[86,87], Christian R Marshall[88], James E Mitchell[89], Richard M Myers[90], Satoshi Okazaki[91], Ikuo Otsuka[66,91], Dalila Pinto[4,5], Abigail Powers[85], Nicolas Ramoz[61], Stephan Ripke[46,92,93], Stefan Roepke[94], Vsevolod Rozanov[95,96], Stephen W Scherer[97,98], Christian Schmahl[50], Marcus Sokolowski[99], Anna Starnawska[100,101,102,103], Michael Strober[104,105], Mei-Hsin Su[56], Laura M Thornton[24], Janet Treasure[106,107], Erin B Ware[108,109], Hunna J Watson[24,110,111], Stephanie H Witt[31], D Blake Woodside[76,77,112,113], Zeynep Yilmaz[24,114,115], Lea Zillich[31], Rolf Adolfsson[116], Ingrid Agartz[117,118,119], Tracy M Air[120], Martin Alda[121,122], Lars Alfredsson[123,124], Adebayo Anjorin[125], Vivek Appadurai[126,127], María Soler Artigas[128,129,130,131], Sandra Van der Auwera[132,133], M Helena Azevedo[134], Nicholas Bass[135], Claiton HD Bau[136,137], Bernhard T Baune[138,139], Frank Bellivier[140,141,142,143], Klaus Berger[144], Joanna M Biernacka[145], Tim B Bigdeli[3,146], Elisabeth B Binder[85,147], Michael Boehnke[148], Marco P Boks[149], Rosa Bosch[128,129,150], David L Braff[151], Richard Bryant[152], Monika Budde[153], Enda M Byrne[13,154], Wiepke Cahn[155], Miguel Casas[128,129,131,150], Enrique Castelao[30], Jorge A Cervilla[156], Boris Chaumette[157,158,159], Sven Cichon[160,161,162,163], Aiden Corvin[164], Nicholas Craddock[165], David Craig[166], Franziska Degenhardt[163], Srdjan Djurovic[167,168], Ayman H Fanous[3,146], Jerome C Foo[169], Andreas J Forstner[160,163,170], Mark Frye[171], Justine M Gatt[14,152], Pablo V Gejman[172,173], Ina Giegling[174,175], Hans J Grabe[132,133], Melissa J Green[14,176], Eugenio H Grevet[177,178], Maria Grigoroiu-Serbanescu[179], Blanca Gutierrez[180], Jose Guzman-Parra[181], Steven P Hamilton[182], Marian L Hamshere[165], Annette M Hartmann[174], Joanna Hauser[183], Stefanie Heilmann-Heimbach[163], Per Hoffmann[161,162,163], Marcus Ising[184], Ian Jones[165], Lisa A Jones[185], Lina Jonsson[186], René S Kahn[5,187], John R Kelsoe[151,188], Kenneth S Kendler[3], Stefan Kloiber[75,184,189], Karestan C Koenen[92,190,191], Manolis Kogevinas[192], Bettina Konte[174], Marie-Odile Krebs[157,158,159], Mikael Landén[22,193], Jacob Lawrence[194], Marion Leboyer[195,196,197], Phil H Lee[92,93,198], Douglas F Levinson[199], Calwing Liao[200,201], Jolanta Lissowska[202], Susanne Lucae[184], Fermin Mayoral[181], Susan L McElroy[203], Patrick McGrath[204], Peter McGuffin[8], Andrew McQuillin[135], Divya Mehta[205,206], Ingrid Melle[20,207], Yuri Milaneschi[208], Philip B Mitchell[176], Esther Molina[209], Gunnar Morken[210,211], Preben Bo Mortensen[101,114,127,212], Bertram Müller-Myhsok[147,213,214], Caroline Nievergelt[151], Vishwajit Nimgaonkar[215], Markus M Nöthen[163], Michael C O'Donovan[165], Roel A Ophoff[68,216], Michael J Owen[165], Carlos Pato[217,217], Michele T Pato[218], Brenda WJH Penninx[219], Jonathan Pimm[135], James B Potash[220], Robert A Power[8,221,222], Martin Preisig[30], Digby Quested[223], Josep Antoni Ramos-Quiroga[128,129,131,150], Andreas Reif[224], Marta Ribasés[128,129,130,131], Vanesa Richarte[128,129,150], Marcella Rietschel[225], Margarita Rivera[8,226], Andrea Roberts[227], Gloria Roberts[176], Guy A Rouleau[228,229], Diego L Rovaris[230], Dan Rujescu[174], Cristina Sánchez-Mora[128,129,130,131], Alan R Sanders[172,173], Peter R Schofield[14,15], Thomas G Schulze[153,169,231,232,233], Laura J Scott[148], Alessandro Serretti[234], Jianxin Shi[235], Stanley I Shyn[236], Lea Sirignano[169], Pamela Sklar[4,5,237], Olav B Smeland[20,21], Jordan W Smoller[92,191,238], Edmund J S Sonuga-Barke[239], Gianfranco Spalletta[240,241], John S Strauss[75,189], Beata Świątkowska[242], Maciej Trzaskowski[13], Ming T Tsuang[243], Gustavo Turecki[244], Laura Vilar-Ribó[128,131], John B Vincent[245], Henry Völzke[246], James TR Walters[165], Cynthia Shannon Weickert[14,176], Thomas W Weickert[14,176], Myrna M Weissman[247,248], Leanne M Williams[249], Naomi R Wray[13,206], Clement C Zai[92,189,190,250,251,252], Esben Agerbo[114,212,253], Anders D Børglum[100,101,102,103], Gerome Breen[7,8], Ditte Demontis[100,101,102,103], Annette Erlangsen[103,254,255,256], Tõnu Esko[257,258], Joel Gelernter[81,82], Stephen J Glatt[259], David M Hougaard[253,260], Hai-Gwo Hwu[261], Po-Hsiu Kuo[54,56], Cathryn M Lewis[8,262], Qingqin S Li[263], Chih-Min Liu[54], Nicholas G Martin[12], Andrew M McIntosh[11], Sarah E Medland[12], Ole Mors[253,264], Merete Nordentoft[253,265], Catherine M Olsen[266], David Porteous[267], Daniel J Smith[268], Eli A Stahl[4,257,269], Murray B Stein[270], Danuta Wasserman[99], Thomas Werge[126,253,271,272], David C Whiteman[266], Virginia Willour[273], the VA Million Veteran Program (MVP), the MVP Suicide Exemplar Workgroup, Suicide Working Group of the Psychiatric Genomics Consortium, Major Depressive Disorder

Working Group of the Psychiatric Genomics Consortium, Bipolar Disorder Working Group of the Psychiatric Genomics Consortium, Schizophrenia Working Group of the Psychiatric Genomics Consortium, Eating Disorder Working Group of the Psychiatric Genomics Consortium, German Borderline Genomics Consortium, Hilary Coon[1,2,274], Jean C Beckham[275,276], Nathan A Kimbrel[275,276], Douglas M Ruderfer[9,277,278]

[1] Huntsman Mental Health Institute, Salt Lake City, UT, USA
[2] University of Utah School of Medicine, Department of Psychiatry, Salt Lake City, UT, USA
[3] Virginia Commonwealth University, Department of Psychiatry, Richmond, VA, USA
[4] Icahn School of Medicine at Mount Sinai, Department of Genetics and Genomic Sciences, New York, NY, USA
[5] Icahn School of Medicine at Mount Sinai, Department of Psychiatry, New York, NY, USA
[6] Duke University Medical Center, Duke Molecular Physiology Institute, Durham, NC, USA
[7] King's College London, National Institute for Health Research (NIHR) Maudsley Biomedical Research Centre at South London and Maudsley NHS Foundation Trust, London, UK
[8] King's College London, Social Genetic and Developmental Psychiatry Centre, London, UK
[9] Vanderbilt University Medical Center, Division of Genetic Medicine, Department of Medicine, Vanderbilt Genetics Institute, Nashville, TN, USA
[10] Yale University School of Medicine, Department of Psychiatry, New Haven, CT, USA
[11] University of Edinburgh, Division of Psychiatry, Edinburgh, UK
[12] QIMR Berghofer Medical Research Institute, Mental Health and Neuroscience Research Program, Brisbane, QLD, Australia
[13] The University of Queensland, Institute for Molecular Bioscience, Brisbane, QLD, Australia
[14] Neuroscience Research Australia, Sydney, NSW, Australia
[15] University of New South Wales, School of Medical Sciences, Sydney, NSW, Australia
[16] University of Pennsylvania Perelman School of Medicine, Department of Psychiatry, Philadelphia, PA, USA
[17] Crescenz VAMC, VISN 4 MIRECC, Philadelphia, PA, USA
[18] The University of Queensland, School of Biomedical Sciences, Faculty of Medicine, Brisbane, QLD, Australia
[19] University of Texas Health Science Center, Department of Psychiatry and Behavioral Sciences, Houston, TX, USA
[20] Oslo University Hospital, Division of Mental Health and Addiction, Oslo, Norway
[21] University of Oslo, NORMENT, Oslo, Norway
[22] Karolinska Institutet, Department of Medical Epidemiology and Biostatistics, Stockholm, Sweden
[23] University of North Carolina at Chapel Hill, Department of Nutrition, Chapel Hill, NC, USA
[24] University of North Carolina at Chapel Hill, Department of Psychiatry, Chapel Hill, NC, USA
[25] Indiana University, Department of Medical & Molecular Genetics, Indianapolis, IN, USA
[26] Indiana University School of Medicine, Biochemistry and Molecular Biology, Indianapolis, IN, USA
[27] Harvard Medical School, Department of Health Care Policy, Boston, MA, USA
[28] Columbia University, Departments of Psychiatry and Radiology, New York, NY, USA
[29] Indiana University School of Medicine, Departments of Psychiatry and Medical and Molecular Genetics, Indianapolis, IN, USA
[30] Lausanne University Hospital and University of Lausanne, Department of Psychiatry, Lausanne, Vaud, Switzerland
[31] Central Institute of Mental Health, Medical Faculty Mannheim, University of Heidelberg, Department of Genetic Epidemiology in Psychiatry, Mannheim, Germany
[32] Uniformed Services University of the Health Sciences, Department of Psychiatry, Bethesda, MD, USA
[33] Duke University Medical Center, Department of Psychiatry and Behavioral Sciences, Durham, NC, USA
[34] Duke University Medical Center, Durham, NC, USA
[35] Durham Veterans Affairs Health Care System, Durham, NC, USA
[36] Miami VA Health Care System, Miami, FL, USA
[37] Durham Veterans Affairs Health Care System, Cooperative Studies Program Epidemiology Center, Durham, NC, USA
[38] Boston VA Health Care System, Boston, MA, USA
[39] Oak Ridge National Laboratory, Oak Ridge, TN, USA
[40] Durham Veterans Affairs Health Care System, VA Health Services Research and Development Center of Innovation to Accelerate Discovery and Practice Transformation, Durham, NC, USA
[41] Argonne National Laboratory, University of Chicago Consortium for Advanced Science and Engineering, Chicago, IL, USA
[42] Los Alamos National Laboratory, Theoretical Division, Los Alamos National Laboratory, Los Alamos, NM, USA

[43] Corporal Michael J. Crescenz VA Medical Center, VISN 4 Mental Illness Research, Education, and Clinical Center, Philadelphia, PA, USA
[44] Perelman School of Medicine, University of Pennsylvania, Department of Psychiatry, Philadelphia, PA, USA
[45] VA Palo Alto Health Care System, VA Program Evaluation and Resource Center, Palo Alto, CA, USA
[46] Charité - Universitätsmedizin Berlin, Department of Psychiatry and Psychotherapy, Berlin, Germany
[47] BioRealm, LLC, Walnut, CA, USA
[48] Oregon Research Institute, Eugene, OR, USA
[49] Perelman School of Medicine at the University of Pennsylvania, Department of Psychiatry, Center for Neurobiology and Behavior, Philadelphia, PA, USA
[50] Central Institute of Mental Health, Medical Faculty Mannheim, University of Heidelberg, Department of Psychosomatic Medicine and Psychotherapy, Mannheim, Germany
[51] ERCPathlight, Baltimore, MD, USA
[52] University of Maryland St. Joseph Medical Center, Baltimore, MD, USA
[53] Children's Hospital of Philadelphia, Center for Applied Genomics, Philadelphia, PA, USA
[54] National Taiwan University Hospital, Department of Psychiatry, Taipei, Taiwan
[55] National Health Research Institutes, Center for Neuropsychiatric Research, Miaoli County, Taiwan
[56] National Taiwan University, Institute of Epidemiology and Preventive Medicine, College of Public Health, Taipei, Taiwan
[57] Utah Department of Health and Human Services, Utah Office of the Medical Examiner, Taylorsville, UT, USA
[58] University of Utah, Department of Pathology, Salt Lake City, UT, USA
[59] University of Minnesota, Department of Psychiatry, Minneapolis, MN, USA
[60] GHU Paris Psychiatrie et Neurosciences, Hôpital Sainte Anne, Paris, France
[61] Université Paris Cité, Institute of Psychiatry and Neuroscience of Paris (IPNP), INSERM U1266, Paris, France
[62] University Hospital Bellvitge-IDIBELL and CIBEROBN, Department of Psychiatry, Barcelona, Spain
[63] Ludwig-Maximilians-University (LMU), Department of Psychiatry and Psychotherapy, Munich, Germany
[64] Schön Klinik Roseneck affiliated with the Medical Faculty of the University of Munich (LMU), Munich, Germany
[65] Columbia University, Department of Biostatistics, New York, NY, USA
[66] Columbia University, Department of Psychiatry, New York, NY, USA
[67] University of Toronto, Department of Surgery, Faculty of Medicine, Toronto, Canada
[68] University of California, Los Angeles, Department of Psychiatry and Biobehavioral Science, Semel Institute, David Geffen School of Medicine, Los Angeles, CA, USA
[69] University of Pennsylvania, The Perelman School of Medicine, Philadelphia, PA, USA
[70] Weill Cornell Medical College, Department of Psychiatry, New York, NY, USA
[71] Yokohama City University Graduate School of Medicine, Department of Psychiatry, Yokohama, Japan
[72] University of California San Diego, Biostatistics Research Center, Herbert Wertheim School of Public Health and Human Longevity Science, La Jolla, CA, USA
[73] Univ Paris-Est-Créteil, INSERM, IMRB, Translational Neuropsychiatry, Fondation FondaMental, Créteil, France
[74] Eating Recovery Center, Denver, CO, USA
[75] Centre for Addiction and Mental Health, Toronto, ON, Canada
[76] University of Toronto, Department of Psychiatry, Toronto, Canada
[77] University of Toronto, Institute of Medical Science, Toronto, Canada
[78] University of California San Diego, Department of Psychiatry, San Diego, CA, USA
[79] Florida State University, Department of Psychology, Tallahassee, FL, USA
[80] Michigan State University, Department of Psychology, Lansing, MI, USA
[81] Veterans Affairs Connecticut Healthcare Center, Department of Psychiatry, West Haven, CT, USA
[82] Yale University School of Medicine, Division of Human Genetics, Department of Psychiatry, New Haven, CT, USA
[83] University Medical Center, Department of Psychiatry and Psychotherapy, Mainz, Germany
[84] The Chicago School of Professional Psychology, Washington DC, Department of Clinical Psychology, Washington, DC, USA
[85] Emory University School of Medicine, Department of Psychiatry and Behavioral Sciences, Atlanta, GA, USA
[86] King Abdullah University of Science and Technology, BESE Division, Thuwal, Saudi Arabia
[87] University of Lausanne-University Hospital of Lausanne (UNIL-CHUV), Department of Psychiatry, Lausanne, Switzerland
[88] The Hospital for Sick Children, Department of Paediatric Laboratory Medicine, Toronto, Canada

[89] University of North Dakota School of Medicine and Health Sciences, Department of Psychiatry and Behavioral Science, Fargo, ND, USA
[90] HudsonAlpha Institute for Biotechnology, Huntsville, AL, USA
[91] Kobe University Graduate School of Medicine, Department of Psychiatry, Kobe, Japan
[92] Broad Institute, Stanley Center for Psychiatric Research, Cambridge, MA, USA
[93] Massachusetts General Hospital, Analytical and Translational Genetics Unit, Boston, MA, USA
[94] Charité - Universitätsmedizin Berlin, Corporate Member of Freie Universität Berlin, Humboldt-Universität zu Berlin, Berlin Institute of Health, Campus Benjamin Franklin, Department of Psychiatry, Berlin, Germany
[95] Saint-Petersburg State University, Department of Psychology, Saint-Petersburg, Russian Federation
[96] V.M. Bekhterev National Medical Research Center for Psychiatry and Neurology, Department of Borderline Disorders and Psychotherapy, Saint-Petersburg, Russian Federation
[97] The Hospital for Sick Children, Department of Genetics and Genomic Biology, Toronto, Canada
[98] University of Toronto, McLaughlin Center, Toronto, Canada
[99] Karolinska Institutet, National Centre for Suicide Research and Prevention of Mental Ill-Health (NASP), LIME, Stockholm, Sweden
[100] Aarhus University, Centre for Genomics and Personalized Medicine, CGPM, Aarhus, Denmark
[101] Aarhus University, Centre for Integrative Sequencing, iSEQ, Aarhus, Denmark
[102] Aarhus University, Department of Biomedicine, Aarhus, Denmark
[103] Aarhus University, The Lundbeck Foundation Initiative for Integrative Psychiatric Research, iPSYCH, Aarhus, Denmark
[104] University of California Los Angeles, David Geffen School of Medicine, Los Angeles, LA, USA
[105] University of California Los Angeles, Department of Psychiatry and Biobehavioral Science, Semel Institute for Neuroscience and Human Behavior, Los Angeles, LA, USA
[106] King's College London, Institute of Psychiatry, Psychology and Neuroscience, Department of Psychological Medicine, London, UK
[107] King's College London and South London and Maudsley National Health Service Foundation Trust, National Institute for Health Research Biomedical Research Centre, London, UK
[108] University of Michigan, Population Studies Center, Institute for Social Research, Ann Arbor, MI, USA
[109] University of Michigan, Survery Research Center, Institute for Social Research, Ann Arbor, MI, USA
[110] Curtin University, School of Psychology, Perth, Western Australia, Australia
[111] The University of Western Australia, Division of Paediatrics, Perth, Western Australia, Australia
[112] University Health Network, Centre for Mental Health, Toronto, Canada
[113] University Health Network, Program for Eating Disorders, Toronto, Canada
[114] Aarhus University, National Centre for Register-Based Research, Aarhus, Denmark
[115] University of North Carolina at Chapel Hill, Department of Genetics, Chapel Hill, NC, USA
[116] Umeå University Medical Faculty, Department of Clinical Sciences, Psychiatry, Umeå, Sweden
[117] Diakonhjemmet Hospital, Department of Psychiatric Research, Oslo, Norway
[118] Karolinska Institutet, Department of Clinical Neuroscience, Centre for Psychiatry Research, Stockholm, Sweden
[119] University of Oslo, NORMENT, Institute of Clinical Medicine, Oslo, Norway
[120] University of Adelaide, Discipline of Psychiatry, Adelaide, SA, Australia
[121] Dalhousie University, Department of Psychiatry, Halifax, NS, Canada
[122] National Institute of Mental Health, Klecany, CZ
[123] Karolinska Institutet, Department of Clinical Neuroscience, Stockholm, Sweden
[124] Karolinska Institutet, Inst of Environmental Medicine, Stockholm, Sweden
[125] Berkshire Healthcare NHS Foundation Trust, Psychiatry, Bracknell, UK
[126] Copenhagen University Hospital, Institute of Biological Psychiatry, Copenhagen Mental Health Services, Copenhagen, Denmark
[127] iPSYCH, The Lundbeck Foundation Initiative for Integrative Psychiatric Research, Copenhagen, Denmark
[128] Hospital Universitari Vall d'Hebron, Department of Psychiatry, Barcelona, Spain
[129] Instituto de Salud Carlos III, Biomedical Network Research Centre on Mental Health (CIBERSAM), Madrid, Spain
[130] University of Barcelona, Department of Genetics, Microbiology & Statistics, Barcelona, Spain
[131] Vall d'Hebron Research Institute (VHIR), Universitat Autònoma de Barcelona, Psychiatric Genetics Unit, Group of Psychiatry, Mental Health and Addiction,, Barcelona, Spain
[132] University Medicine Greifswald, Department of Psychiatry and Psychotherapy, Greifswald, Mecklenburg-

Vorpommern, Germany
[133] German Centre for Neurodegenerative Diseases (DZNE), Partner Site Rostock/Greifswald, Greifswald, Mecklenburg-Vorpommern, Germany
[134] University of Coimbra, Department of Psychiatry, Coimbra, Portugal
[135] University College London, Division of Psychiatry, London, UK
[136] Hospital de Clínicas de Porto Alegre, Laboratory of Developmental Psychiatry, Porto Alegre, RS, Brazil
[137] Universidade Federal do Rio Grande do Sul, Department of Genetics, Porto Alegre, RS, Brazil
[138] University of Melbourne, Department of Psychiatry, Melbourne Medical School, Melbourne, Australia
[139] University of Münster, Department of Psychiatry, Münster, Germany
[140] Assistance Publique - Hôpitaux de Paris, Department of Psychiatry and Addiction Medicine, Paris, France
[141] FondaMental Foundation, Paris Bipolar and TRD Expert Centres, Paris, France
[142] INSERM, UMR-S1144 Team 1 : Biomarkers of relapse and therapeutic response in addiction and mood disorders, Paris, France
[143] Université Paris Cité, Psychiatry, Paris, France
[144] University of Münster, Institute of Epidemiology and Social Medicine, Münster, Nordrhein-Westfalen, Germany
[145] Mayo Clinic, Health Sciences Research, Rochester, MN, USA
[146] State University of New York Downstate Medical Center, Department of Psychiatry and Behavioral Sciences, New York, NY, USA
[147] Max Planck Institute of Psychiatry, Department of Translational Research in Psychiatry, Munich, Germany
[148] University of Michigan, Center for Statistical Genetics and Department of Biostatistics, Ann Arbor, MI, USA
[149] UMC Utrecht Brain Center, Psychiatry, Utrecht, Netherlands
[150] Universitat Autònoma de Barcelona, Department of Psychiatry and Legal Medicine, Barcelona, Spain
[151] University of California San Diego, Department of Psychiatry, La Jolla, CA, USA
[152] University of New South Wales, School of Psychology, Sydney, NSW, Australia
[153] University Hospital, LMU Munich, Institute of Psychiatric Phenomics and Genomics (IPPG), Munich, Germany
[154] The University of Queensland, Child Health Research Centre, Brisbane, QLD, Australia
[155] UMC Utrecht Hersencentrum Rudolf Magnus, Department of Psychiatry, Utrecht, Netherlands
[156] University of Granada, Mental Health Unit, Department of Psychiatry, Faculty of Medicine, Granada University Hospital Complex, Granada, Spain
[157] CNRS GDR 3557, Institut de Psychiatrie, Paris, France
[158] GHU Paris Psychiatrie et Neurosciences, Department of Evaluation, Prevention and Therapeutic innovation, Paris, France
[159] Université de Paris, Institute of Psychiatry and Neuroscience of Paris (IPNP), INSERM U1266, Team Pathophysiology of psychiatric diseases, Paris, France
[160] Research Centre Jülich, Institute of Neuroscience and Medicine (INM-1), Jülich, Germany
[161] University Hospital Basel, Institute of Medical Genetics and Pathology, Basel, Switzerland
[162] University of Basel, Department of Biomedicine, Basel, Switzerland
[163] University of Bonn, School of Medicine & University Hospital Bonn, Institute of Human Genetics, Bonn, Germany
[164] Trinity College Dublin, Neuropsychiatric Genetics Research Group, Dept of Psychiatry and Trinity Translational Medicine Institute, Dublin, Ireland
[165] Cardiff University, Medical Research Council Centre for Neuropsychiatric Genetics and Genomics, Division of Psychological Medicine and Clinical Neurosciences, Cardiff, UK
[166] University of Southern California, Department of Translational Genomics, Pasadena, CA, USA
[167] Oslo University Hospital, Department of Medical Genetics, Oslo, Norway
[168] University of Bergen, NORMENT, KG Jebsen Centre for Psychosis Research, Department of Clinical Science, Bergen, Norway
[169] Central Institute of Mental Health, Medical Faculty Mannheim, Heidelberg University, Department of Genetic Epidemiology in Psychiatry, Mannheim, Germany
[170] University of Marburg, Centre for Human Genetics, Marburg, Germany
[171] Mayo Clinic, Department of Psychiatry & Psychology, Rochester, MN, USA
[172] NorthShore University HealthSystem, Department of Psychiatry and Behavioral Sciences, Evanston, IL, USA
[173] University of Chicago, Department of Psychiatry and Behavioral Neuroscience, Chicago, IL, USA
[174] Medical University of Vienna, Department of Psychiatry and Psychotherapy, Vienna, Austria
[175] University of Munich, Department of Psychiatry, Munich, Germany

[176] University of New South Wales, School of Psychiatry, Sydney, NSW, Australia
[177] Hospital de Clínicas de Porto Alegre, ADHD Outpatient Program, Adult Division, Porto Alegre, RS, Brazil
[178] Universidade Federal do Rio Grande do Sul, Department of Psychiatry, Porto Alegre, RS, Brazil
[179] Alexandru Obregia Clinical Psychiatric Hospital, Biometric Psychiatric Genetics Research Unit, Bucharest, Romania
[180] University of Granada, Department of Psychiatry, Faculty of Medicine and Biomedical Research Centre (CIBM), Granada, Spain
[181] University Regional Hospital. Biomedicine Institute (IBIMA), Mental Health Department, Málaga, Spain
[182] Kaiser Permanente Northern California, Psychiatry, San Francisco, CA, USA
[183] Poznan University of Medical Sciences, Psychiatric Genetics, Department of Psychiatry, Poznan, Poland
[184] Max Planck Institute of Psychiatry, Munich, Germany
[185] University of Worcester, Department of Psychological Medicine, Worcester, UK
[186] University of Gothenburg, Department of Psychiatry and Neuroscience, Gothenburg, Sweden
[187] UMC Utrecht Brain Center Rudolf Magnus, Psychiatry, Utrecht, Netherlands
[188] University of California San Diego, Institute for Genomic Medicine, La Jolla, CA, USA
[189] University of Toronto, Department of Psychiatry, Toronto, ON, Canada
[190] Harvard TH Chan School of Public Health, Department of Epidemiology, Boston, MA, USA
[191] Massachusetts General Hospital, Department of Psychiatry, Boston, MA, USA
[192] Center for Research in Environmental Epidemiology (CREAL), Barcelona, Spain
[193] University of Gothenburg, Institute of Neuroscience and Physiology, Gothenburg, Sweden
[194] North East London NHS Foundation Trust, Psychiatry, Ilford, UK
[195] Univ Paris Est Créteil, INSERM, AP-HP, IMRB, Translational Neuropsychiatry, DMU IMPACT, FHU ADAPT, Fondation FondaMental, Créteil, France
[196] INSERM, Paris, France
[197] Université Paris Est, Faculté de Médecine, Créteil, France
[198] Massachusetts General Hospital, Psychiatric and Neurodevelopmental Genetics Unit, Boston, MA, USA
[199] Stanford University, Psychiatry & Behavioral Sciences, Stanford, CA, USA
[200] Broad Institute of MIT and Harvard, Stanley Center for Psychiatric Research, Cambridge, MA, USA
[201] Massachusetts General Hospital, Analytical and Translational Genetics Unit, Cambridge, MA, USA
[202] M. Sklodowska-Curie Cancer Center and Institute of Oncology, Cancer Epidemiology and Prevention, Warsaw, Poland
[203] Lindner Center of HOPE, Research Institute, Mason, OH, USA
[204] Columbia University College of Physicians and Surgeons, Psychiatry, New York, NY, USA
[205] Queensland University of Technology, School of Psychology and Counseling, Brisbane, QLD, Australia
[206] The University of Queensland, Queensland Brain Institute, Brisbane, QLD, Australia
[207] University of Oslo, Institute of Clinical Medicine, Division of Mental Health and Addiction, Oslo, Norway
[208] Amsterdam UMC, Vrije Universiteit and GGZ inGeest, Department of Psychiatry, Amsterdam, Netherlands
[209] University of Granada, Department of Nursing, Faculty of Health Sciences and Biomedical Research Centre (CIBM), Granada, Spain
[210] Norwegian University of Science and Technology - NTNU, Mental Health, Faculty of Medicine and Health Sciences, Trondheim, Norway
[211] St Olavs University Hospital, Psychiatry, Trondheim, Norway
[212] Aarhus University, Centre for Integrated Register-based Research, Aarhus, Denmark
[213] Munich Cluster for Systems Neurology (SyNergy), Munich, Germany
[214] University of Liverpool, Liverpool, UK
[215] University of Pittsburgh, Psychiatry and Human Genetics, Pittsburgh, PA, USA
[216] Erasmus University Medical Center, Psychiatry, Rotterdam, Netherlands
[217] Rutgers University, RWJMS,NJMS,UBHC, Pisctatway, NJ, USA
[218] Rutgers University, RWJMS,NJMS, Pisctatway, NJ, USA
[219] Amsterdam UMC, Vrije Universiteit, Department of Psychiatry and Amsterdam Neuroscience, Amsterdam, Netherlands
[220] Johns Hopkins University School of Medicine, Psychiatry, Baltimore, MD, USA
[221] BioMarin Pharmaceuticals, Genetics, London, UK
[222] University of Oxford, St Edmund Hall, Oxford, UK

[223] University of Oxford, Department of Psychiatry, Oxford, UK
[224] University Hospital Frankfurt, Department of Psychiatry, Psychosomatic Medicine and Psychotherapy, Frankfurt, Germany
[225] Central Institute of Mental Health, Medical Faculty Mannheim, Heidelberg University, Department of Genetic Epidemiology in Psychiatry, Mannheim, Baden-Württemberg, Germany
[226] University of Granada, Department of Biochemistry and Molecular Biology II and Institute of Neurosciences, Biomedical Research Centre (CIBM), Granada, Spain
[227] Harvard TH Chan School of Public Health, Department of Environmental Health, Boston, MA, USA
[228] McGill University, Faculty of Medicine, Department of Neurology and Neurosurgery, Montreal, QC, Canada
[229] Montreal Neurological Institute and Hospital, Montreal, QC, Canada
[230] Instituto de Ciencias Biomedicas Universidade de Sao Paulo, Department of Physiology and Biophysics, São Paulo, SP, Brazil
[231] Johns Hopkins University School of Medicine, Department of Psychiatry and Behavioral Sciences, Baltimore, MD, USA
[232] National Institute of Mental Health, Human Genetics Branch, Intramural Research Program, Bethesda, MD, USA
[233] University Medical Center Göttingen, Department of Psychiatry and Psychotherapy, Göttingen, Germany
[234] University of Bologna, Department of Biomedical and NeuroMotor Sciences, Bologna, Italy
[235] National Cancer Institute, Division of Cancer Epidemiology and Genetics, Bethesda, MD, USA
[236] Kaiser Permanente Washington, Behavioral Health Services, Seattle, WA, USA
[237] Icahn School of Medicine at Mount Sinai, Department of Neuroscience, New York, NY, USA
[238] Massachusetts General Hospital, Psychiatric and Neurodevelopmental Genetics Unit (PNGU), Boston, MA, USA
[239] King's College London, Institute of Psychology, Psychiatry & Neuroscience, London, UK
[240] Baylor College of Medicine, Houston, Menninger Department of Psychiatry and Behavioral Sciences, Houston, TX, USA
[241] IRCCS Santa Lucia Foundation, Rome, Laboratory of Neuropsychiatry, Rome, Italy
[242] Nofer Institute of Occupational Medicine, Department of Environmental Epidemiology, Lodz, Poland
[243] University of California, San Diego, Center for Behavioral Genomics, Department of Psychiatry, La Jolla, CA, USA
[244] McGill University, Department of Psychiatry, Montreal, QC, Canada
[245] Centre for Addiction and Mental Health, Molecular Brain Science, Toronto, ON, Canada
[246] University Medicine Greifswald, Institute for Community Medicine, Greifswald, Mecklenburg-Vorpommern, Germany
[247] Columbia University College of Physicians and Surgeons, New York, NY, USA
[248] New York State Psychiatric Institute, Division of Translational Epidemiology, New York, NY, USA
[249] Stanford University, Department of Psychiatry and Behavioral Sciences, Stanford, CA, USA
[250] University of Toronto, Institute of Medical Science, Toronto, ON, Canada
[251] Centre for Addiction and Mental Health, Molecular Brain Science, Campbell Family Mental Health Research Institute, Toronto, ON, Canada
[252] University of Toronto, Laboratory Medicine and Pathobiology, Toronto, ON, Canada
[253] iPSYCH, The Lundbeck Foundation Initiative for Integrative Psychiatric Research, Aarhus, Denmark
[254] Australian National University, Center of Mental Health Research, Canberra, Australia
[255] Johns Hopkins Bloomberg School of Public Health, Department of Mental Health, Baltimore, MD, USA
[256] Mental Health Centre Copenhagen, Danish Research Institute for Suicide Prevention, Copenhagen, Denmark
[257] Broad Institute, Program in Medical and Population Genetics, Cambridge, MA, USA
[258] University of Tartu, Estonian Genome Center, Institute of Genomics, Tartu, Estonia
[259] SUNY Upstate Medical University, Department of Psychiatry and Behavioral Sciences, Syracuse, NY, USA
[260] Statens Serum Institut, Center for Neonatal Screening, Department for Congenital Disorders, Copenhagen, Denmark
[261] National Taiwan University Hospital and College of Medicine, Department of Psychiatry, Taipei, Taiwan
[262] King's College London, Department of Medical & Molecular Genetics, London, UK
[263] Janssen Research & Development, LLC, Neuroscience, Titusville, NJ, USA
[264] Aarhus University Hospital, Risskov, Psychosis Research Unit, Aarhus, Denmark
[265] Copenhagen University Hospital, Mental Health Center Copenhagen, Copenhagen, Denmark
[266] QIMR Berghofer Medical Research Institute, Department of Population Health, Brisbane, QLD, Australia
[267] University of Edinburgh, Institute for Genetics and Molecular Medicine, Edinburgh, UK

[268] University of Edinburgh, Centre for Clinical Brain Sciences, Edinburgh, UK
[269] Regeneron Genetics Center, Analytical Genetics and Data Science, Tarrytown, NY, USA
[270] University of California San Diego, Department of Psychiatry and School of Public Health, La Jolla, CA, USA
[271] University of Copenhagen, Department of Clinical Medicine, Copenhagen, Denmark
[272] University of Copenhagen, Lundbeck Foundation GeoGenetics Centre, GLOBE Institute,, Copenhagen, Denmark
[273] University of Iowa, Department of Psychiatry, Iowa City, IA, USA
[274] University of Utah School of Medicine, Biomedical Informatics, Salt Lake City, UT, USA
[275] Durham Veterans Affairs Health Care System, VISN 6 Mid-Atlantic Mental Illness Research, Education, and Clinical Center, Durham, NC, USA
[276] Duke University School of Medicine, Department of Psychiatry and Behavioral Sciences, Durham, NC, USA
[277] Vanderbilt University Medical Center, Department of Biomedical Informatics, Nashville, TN, USA
[278] Vanderbilt University Medical Center, Department of Psychiatry and Behavioral Sciences, Nashville, TN, USA

MVP Suicide Exemplar Workgroup

Silvia Crivelli, Ph.D. (Lawrence Berkeley National Laboratory), Michelle F. Dennis, B.A. (Durham Veterans Affairs Health Care System & Duke University School of Medicine), Phillip D. Harvey, Ph.D. (University of Miami Miller School of Medicine, Miami, FL), Bruce W. Carter (VA Medical Center), Jennifer E. Huffman, Ph.D. (Massachusetts Veterans Epidemiology Research and Information Center, VA Boston Healthcare System), Daniel Jacobson, Ph.D. (Oak Ridge National Laboratory), Ravi Madduri, Ph.D. (Argonne National Laboratory), Maren K. Olsen, Ph.D. (Duke University School of Medicine), and John Pestian, Ph.D. (Oak Ridge National Laboratory).

Veterans Administration Million Veteran Program (MVP)

J. Michael Gaziano, M.D., M.P.H. (co-chair, VA Boston Healthcare System), Sumitra Muralidhar, Ph.D. (co-chair, U.S. Department of Veterans Affairs), Rachel Ramoni, D.M.D., Sc.D. (U.S. Department of Veterans Affairs), Jean Beckham, Ph.D. (Durham VA Medical Center), Kyong-Mi Chang, M.D. (Philadelphia VA Medical Center), Christopher J. O'Donnell, M.D., M.P.H. (VA Boston Healthcare System), Philip S. Tsao, Ph.D. (VA Palo Alto Health Care System), James Breeling, M.D. (Ex-Officio, U.S. Department of Veterans Affairs), Grant Huang, Ph.D. (Ex-Officio, U.S. Department of Veterans Affairs), and J.P. Casas Romero, M.D., Ph.D. (Ex-Officio, VA Boston Healthcare System). MVP Program Office: Sumitra Muralidhar, Ph.D., and Jennifer Moser, Ph.D., both of U.S. Department of Veterans Affairs. MVP Recruitment/Enrollment: Recruitment/Enrollment Director/Deputy Director, Boston—Stacey B. Whitbourne, Ph.D., Jessica V. Brewer, M.P.H. (VA Boston Healthcare System). MVP Coordinating Centers: Clinical Epidemiology Research Center (CERC), West Haven—Mihaela Aslan, Ph.D. (West Haven VA Medical Center). Cooperative Studies Program Clinical Research Pharmacy Coordinating Center, Albuquerque—Todd Connor, Pharm.D., Dean P. Argyres, B.S., M.S. (New Mexico VA Health Care System). Genomics Coordinating Center, Palo Alto—Philip S. Tsao, Ph.D. (VA Palo Alto Health Care System). MVP Boston Coordinating Center, Boston—J. Michael Gaziano, M.D., M.P.H. (VA Boston Healthcare System). MVP Information Center, Canandaigua—Brady Stephens, M.S. (Canandaigua VA Medical Center). VA Central Biorepository, Boston—Mary T. Brophy, M.D., M.P.H., Donald E. Humphries, Ph.D., Luis E. Selva, Ph.D. (VA Boston Healthcare System). MVP Informatics, Boston—Nhan Do, M.D., Shahpoor Shayan (VA Boston Healthcare System). MVP Data Operations/Analytics, Boston—Kelly Cho, Ph.D. (VA Boston Healthcare System). MVP Science: Science Operations—Christopher J. O'Donnell, M.D., M.P.H. (VA Boston Healthcare System). Genomics Core— Christopher J. O'Donnell, M.D., M.P.H., Saiju Pyarajan, Ph.D. (VA Boston Healthcare System), Philip S. Tsao, Ph.D. (VA Palo Alto Health Care System). Phenomics Core—Kelly Cho, M.P.H., Ph.D. (VA Boston Healthcare System). Data and Computational Sciences—Saiju Pyarajan, Ph.D. (VA Boston Healthcare System). Statistical Genetics—Elizabeth Hauser, Ph.D. (Durham VA Medical Center). Yan Sun, Ph.D. (Atlanta VA Medical Center). Hongyu Zhao, Ph.D. (West Haven VA Medical Center. Current MVP Local Site Investigators: Peter Wilson, M.D. (Atlanta VA Medical Center); Rachel McArdle, Ph.D. (Bay Pines VA Healthcare System); Louis Dellitalia, M.D. (Birmingham VA Medical Center); Kristin Mattocks, Ph.D., M.P.H. (Central Western Massachusetts Healthcare System); John Harley, M.D., Ph.D. (Cincinnati VA Medical Center); Clement J. Zablocki (VA Medical Center); Jeffrey Whittle, M.D., M.P.H.; Frank Jacono, M.D. (VA Northeast Ohio Healthcare System); Jean Beckham, Ph.D. (Durham VA Medical Center); Edith Nourse Rogers Memorial Veterans Hospital; Salvador Gutierrez, M.D. (Edward Hines, Jr. VA Medical Center); Gretchen Gibson, D.D.S., M.P.H. (Veterans Health Care System of the Ozarks); Kimberly Hammer, Ph.D. (Fargo VA Health Care System); Laurence Kaminsky, Ph.D. (VA Health Care Upstate New York); Gerardo Villareal, M.D. (New Mexico VA Health Care System); Scott Kinlay, M.B.B.S., Ph.D. (VA Boston Healthcare System); Junzhe Xu, M.D. (VA Western New York Healthcare System); Mark Hamner, M.D. (Ralph H. Johnson VA Medical Center); Roy Mathew, M.D. (Columbia VA

Health Care System); Sujata Bhushan, M.D. (VA North Texas Health Care System); Pran Iruvanti, DO, Ph.D. (Hampton VA Medical Center); Michael Godschalk, M.D. (Richmond VA Medical Center); Zuhair Ballas, M.D. (Iowa City VA Health Care System); Douglas Ivins, M.D. (Eastern Oklahoma VA Health Care System); Stephen Mastorides, M.D. (James A. Haley Veterans' Hospital); Jonathan Moorman, M.D., Ph.D. (James H. Quillen VA Medical Center); Saib Gappy, M.D. (John D. Dingell VA Medical Center); Jon Klein, M.D., Ph.D. (Louisville VA Medical Center); Nora Ratcliffe, M.D. (Manchester VA Medical Center); Hermes Florez, M.D., Ph.D. (Miami VA Health Care System); Olaoluwa Okusaga, M.D. (Michael E. DeBakey VA Medical Center); Maureen Murdoch, M.D., M.P.H. (Minneapolis VA Health Care System); Peruvemba Sriram, M.D. (N FL/S GA Veterans Health System); Shing Shing Yeh, Ph.D., M.D. (Northport VA Medical Center); Neeraj Tandon, M.D. (Overton Brooks VA Medical Center); Darshana Jhala, M.D. (Philadelphia VA Medical Center); Samuel Aguayo, M.D. (Phoenix VA Health Care System); David Cohen, M.D. (Portland VA Medical Center); Satish Sharma, M.D. (Providence VA Medical Center); Suthat Liangpunsakul, M.D., M.P.H. (Richard Roudebush VA Medical Center); Kris Ann Oursler, M.D. (Salem VA Medical Center); Mary Whooley, M.D. (San Francisco VA Health Care System); Sunil Ahuja, M.D. (South Texas Veterans Health Care System); Joseph Constans, Ph.D. (Southeast Louisiana Veterans Health Care System); Paul Meyer, M.D., Ph.D. (Southern Arizona VA Health Care System); Jennifer Greco, M.D. (Sioux Falls VA Health Care System); Michael Rauchman, M.D. (St. Louis VA Health Care System); Richard Servatius, Ph.D. (Syracuse VA Medical Center); Melinda Gaddy, Ph.D. (VA Eastern Kansas Health Care System); Agnes Wallbom, M.D., M.S. (VA Greater Los Angeles Health Care System); Timothy Morgan, M.D. (VA Long Beach Healthcare System); Todd Stapley, D.O. (VA Maine Healthcare System); Scott Sherman, M.D., M.P.H. (VA New York Harbor Healthcare System); George Ross, M.D. (VA Pacific Islands Health Care System); Philip Tsao, Ph.D. (VA Palo Alto Health Care System); Patrick Strollo Jr., M.D. (VA Pittsburgh Health Care System); Edward Boyko, M.D. (VA Puget Sound Health Care System); Laurence Meyer, M.D., Ph.D. (VA Salt Lake City Health Care System); Samir Gupta, M.D., M.S.C.S. (VA San Diego Healthcare System); Mostaqul Huq, Pharm.D., Ph.D. (VA Sierra Nevada Health Care System); Joseph Fayad, M.D. (VA Southern Nevada Healthcare System); Adriana Hung, M.D., M.P.H. (VA Tennessee Valley Healthcare System); Jack Lichy, M.D., Ph.D. (Washington, DC VA Medical Center); Robin Hurley, M.D. (W.G., Bill Hefner VA Medical Center); Brooks Robey, M.D. (White River Junction VA Medical Center); and Robert Striker, M.D., Ph.D. (William S. Middleton Memorial Veterans Hospital).
