# Supplementary material for: Connecting genomic results for psychiatric disorders to human brain cell types and regions reveals convergence with functional connectivity
Source: Nat Commun. 2025 Jan 4;16:395. doi: 10.1038/s41467-024-55611-1 (PMC11700164; doi:10.1038/s41467-024-55611-1)
Supplement: Supplementary file 2 — Reporting Summary [file 41467_2024_55611_MOESM2_ESM.pdf]

nature portfolio

Corresponding author(s): DAPR NCOMMS-24-49193A

Last updated by author(s): Nov 15, 2024

Reporting Summary

Nature Portfolio wishes to improve the reproducibility of the work that we publish. This form provides structure for consistency and transparency in reporting. For further information on Nature Portfolio policies, see our Editorial Policies and the Editorial Policy Checklist.

Statistics

For all statistical analyses, confirm that the following items are present in the figure legend, table legend, main text, or Methods section.

n/a	Confirmed	
☐	☒	The exact sample size (*n*) for each experimental group/condition, given as a discrete number and unit of measurement
☐	☒	A statement on whether measurements were taken from distinct samples or whether the same sample was measured repeatedly
☐	☒	The statistical test(s) used AND whether they are one- or two-sided<br>*Only common tests should be described solely by name; describe more complex techniques in the Methods section.*
☐	☒	A description of all covariates tested
☐	☒	A description of any assumptions or corrections, such as tests of normality and adjustment for multiple comparisons
☐	☒	A full description of the statistical parameters including central tendency (e.g. means) or other basic estimates (e.g. regression coefficient) AND variation (e.g. standard deviation) or associated estimates of uncertainty (e.g. confidence intervals)
☐	☒	For null hypothesis testing, the test statistic (e.g. *F*, *t*, *r*) with confidence intervals, effect sizes, degrees of freedom and *P* value noted<br>*Give P values as exact values whenever suitable.*
☒	☐	For Bayesian analysis, information on the choice of priors and Markov chain Monte Carlo settings
☐	☒	For hierarchical and complex designs, identification of the appropriate level for tests and full reporting of outcomes
☐	☒	Estimates of effect sizes (e.g. Cohen's *d*, Pearson's *r*), indicating how they were calculated

Our web collection on statistics for biologists contains articles on many of the points above.

Software and code

Policy information about availability of computer code

Data collection	Data was publicly available. GWAS summary statistics are listed in Supplementary Table S1 with details in the Method
Data analysis	https://github.com/Hjerling-Leffler-Lab/TDEP-sLDSC

For manuscripts utilizing custom algorithms or software that are central to the research but not yet described in published literature, software must be made available to editors and reviewers. We strongly encourage code deposition in a community repository (e.g. GitHub). See the Nature Portfolio guidelines for submitting code & software for further information.

Data

Policy information about availability of data

All manuscripts must include a data availability statement. This statement should provide the following information, where applicable:

- Accession codes, unique identifiers, or web links for publicly available datasets
- A description of any restrictions on data availability
- For clinical datasets or third party data, please ensure that the statement adheres to our policy

Source data are provided with this paper. The data generated in this study are provided in the Supplementary Information and Source Data files.

Research involving human participants, their data, or biological material

Policy information about studies with human participants or human data. See also policy information about sex, gender (identity/presentation), and sexual orientation and race, ethnicity and racism.

Reporting on sex and gender	The genomic analyses used GWAS summary statistics where sex was a covariate. The fMRI analysis matched case and control by age and sex.
Reporting on race, ethnicity, or other socially relevant groupings	We used summary level genetic and transcriptomic data. The GWAS results were based on the populations with European ancestry, where the statistical power was the highest.
Population characteristics	See above
Recruitment	N/A
Ethics oversight	N/A

Note that full information on the approval of the study protocol must also be provided in the manuscript.

Field-specific reporting

Please select the one below that is the best fit for your research. If you are not sure, read the appropriate sections before making your selection.

☒ Life sciences ☐ Behavioural & social sciences ☐ Ecological, evolutionary & environmental sciences

For a reference copy of the document with all sections, see nature.com/documents/nr-reporting-summary-flat.pdf

Life sciences study design

All studies must disclose on these points even when the disclosure is negative.

Sample size	We used public data for analysis. The GWAS sample size were on the thousand scale and summarised in Table S1. The snRNA-seq data was based on 4 donors, and the fMRI analyses were based on two independent public samples with sample sizes 92 and 108.
Data exclusions	N/A
Replication	The fMRI analysis was performed in two independent datasets.
Randomization	N/A
Blinding	N/A

Reporting for specific materials, systems and methods

We require information from authors about some types of materials, experimental systems and methods used in many studies. Here, indicate whether each material, system or method listed is relevant to your study. If you are not sure if a list item applies to your research, read the appropriate section before selecting a response.

Materials & experimental systems

n/a	Involved in the study	
☒	☐	Antibodies
☒	☐	Eukaryotic cell lines
☒	☐	Palaeontology and archaeology
☒	☐	Animals and other organisms
☒	☐	Clinical data
☒	☐	Dual use research of concern
☒	☐	Plants

Methods

n/a	Involved in the study	
☒	☐	ChIP-seq
☒	☐	Flow cytometry
☐	☒	MRI-based neuroimaging

Plants

Seed stocks	N/A
Novel plant genotypes	N/A
Authentication	N/A

Magnetic resonance imaging

Experimental design

Design type	We used publicly available, pre-processed resting-state fMRI data, based on matched case-contorl setting.
Design specifications	N/A
Behavioral performance measures	resting-state fMRI

Acquisition

Imaging type(s)	resting-state fMRI
Field strength	3T
Sequence & imaging parameters	dataset 1: https://www.openfmri.org/dataset/ds000030/<br>dataset 2: https://fcon_1000.projects.nitrc.org/indi/retro/cobre.html
Area of acquisition	Whole brain

Diffusion MRI ☐ Used ☒ Not used

Preprocessing

Preprocessing software	We used publicly available, pre-processed data. Info can be found here: https://f1000research.com/articles/6-1262/v2 and COBRE (nitrc.org)
Normalization	see Method of paper
Normalization template	MINI
Noise and artifact removal	see Method of the paper
Volume censoring	see Method of the paper

Statistical modeling & inference

Model type and settings	N/A
Effect(s) tested	N/A

Specify type of analysis: ☐ Whole brain ☐ ROI-based ☐ Both

Statistic type for inference<br>(See Eklund et al. 2016)	N/A
Correction	N/A

Models & analysis

n/a | Involved in the study

- [ ] | [x] Functional and/or effective connectivity
- [ ] | [x] Graph analysis
- [ ] | [x] Multivariate modeling or predictive analysis

Functional and/or effective connectivity	functional connectivity inferred from resting-state fMRI
Graph analysis	We represent functional connectivity as a graph, with nodes corresponding to brain regions (ROIs) and edges based on the Pearson correlation of the BOLD signal between them.
Multivariate modeling and predictive analysis	We apply neural networks (MLP) to predict outcomes using the functional connectivity graph as input.
